# Real-world outcomes of mepolizumab for the treatment of severe eosinophilic asthma in Canada: an observational study

**DOI:** 10.1186/s13223-023-00863-7

**Published:** 2024-02-04

**Authors:** Kenneth R. Chapman, Kathryn Cogger, Erin Arthurs, Callahan LaForty, Shane Golden, Bradley Millson, Koyo Usuba, Christopher Licskai

**Affiliations:** 1 Asthma & Airway Centre, University Health Network, Room 7-451 EW, 399 Bathurst Street, Toronto, ON M5T 2S8 Canada; 2 GSK, Mississauga, ON Canada; 3 IQVIA, Toronto, ON Canada; 4 IQVIA, Montreal, QC Canada; 5 IQVIA, Ottawa, ON Canada; 6 Western University, London, ON Canada

**Keywords:** Mepolizumab, Severe asthma, Eosinophilic, Real-world, Canada, Costs, Healthcare resource utilization, Exacerbations, ICES, Patient support program

## Abstract

**Background:**

Mepolizumab, the first widely available anti-interleukin 5 biologic, targets eosinophilic inflammation and has been shown in clinical trials to reduce exacerbations, oral corticosteroid dependence, and healthcare utilization in patients with severe asthma. The impact of mepolizumab in a real-world, publicly funded healthcare setting is unknown. The objective of this study was to describe the demographics and clinical characteristics of real-world patients receiving mepolizumab, and to compare asthma-related outcomes and associated asthma-related costs before and during mepolizumab use.

**Methods:**

This retrospective, observational study in Ontario, Canada, included patients initiating mepolizumab between February 2016 and March 2019. Patients were identified using the mepolizumab patient support program and linked to the Institute for Clinical Evaluative Sciences database of publicly accessed healthcare. Patient outcomes were obtained for 12 months pre- and post-mepolizumab initiation and compared.

**Results:**

A total of 275 patients were enrolled in the overall patient support program cohort (mean [standard deviation] age 57.6 [13.5] years, mean [standard deviation] of the median per-patient eosinophil count 540.4 [491.9] cells/μL). Mepolizumab was associated with reductions in asthma exacerbations (46.1%, *P* < 0.001) and in the number of asthma-related visits to general practitioners (40.2%, *P* < 0.001), specialists (27.2%, *P* < 0.001), and emergency departments (52.1%, *P* < 0.001). Associated costs were significantly lower post- versus pre-mepolizumab for asthma-related general practitioner and specialist visits, and for all-cause emergency department visits and hospital admissions.

**Conclusions:**

In a real-world population of Canadian patients with severe asthma with an eosinophilic phenotype, the use of mepolizumab within a patient support program reduced asthma exacerbations and decreased asthma-related healthcare resource utilization and associated costs.

**Supplementary Information:**

The online version contains supplementary material available at 10.1186/s13223-023-00863-7.

## Introduction

Severe asthma represents less than 10% of the total asthma population but the care of such patients accounts for a disproportionate share of asthma care costs. In the United States (US), the annual direct cost per patient is up to US$9,175 for severe asthma [1], whereas in Quebec, Canada, a 2019 study reported annual per-patient costs of CAD$11,946 and CAD$2,884 for very severe and uncontrolled asthma, respectively [2]. In the US, the cost of treating severe asthma was substantially greater than treating mild-to-moderate asthma [1]. Costs were related to outpatient visits, emergency department (ED) visits, and hospitalizations, with a loss of productivity associated with missed work or school days.

Up to 80% of severe asthma with an eosinophilic phenotype (SA-EP) is associated with type 2 inflammation, for which eosinophils are a key effector cell [3, 4]. Mepolizumab is an anti-interleukin 5 (IL5) biologic that reduces the number of circulating eosinophils by blocking IL5 signaling. In randomized trials, mepolizumab has been shown to reduce clinically significant exacerbations (CSEs; defined as asthma worsening requiring systemic corticosteroids administration for ≥ 3 days and/or ED visit and/or hospitalization) in patients with SA-EP by up to 53% versus placebo [5].

A global, prospective, observational cohort study, REALITI-A, showed that real-world rates of CSEs were reduced by 71% with mepolizumab treatment, as were exacerbations requiring hospitalizations and/or ED visits (76% reduction), while median daily oral corticosteroid (OCS) dose (in patients using maintenance OCS) decreased by 75% (10 mg/day to 2.5 mg/day) after 1 year of treatment [6]. Further studies have supported these findings, including the real-world REDES study in Spain, where mepolizumab reduced CSEs in patients with SA-EP by up to 77.5% compared to baseline [7–9]. Whilst a strength of REALITI-A was its multi-national study design, evaluation in a variety of real-world populations within different healthcare systems is invaluable. For example, the REALITI-A findings are heavily influenced by the impact of concomitant daily OCS use, a management strategy employed more commonly in Europe than in North America [6]. Additionally, the background healthcare delivery system can influence the apparent impact of biologic therapies in severe asthma due to differences in the availability of healthcare and background inhaled medications [10, 11].

Canada has a publicly funded universal healthcare system that is implemented at the provincial level. In Ontario, the most populous province in Canada, all health services are provided within the public system and can be analyzed using the ICES (formerly known as the Institute for Clinical and Evaluative Sciences [ICES]) database [12]. Furthermore, the Ontario Drug Benefit (ODB) program provides coverage for more than 5,000 drug products to individuals who qualify for reduced prescription drug costs [13]. Ontario residents who qualify include those aged ≥ 65 years, on social assistance, residing in special or long-term care homes, receiving professional home care services, and/or registrants in the Trillium Drug Program [13].

Mepolizumab was first approved in Canada in December 2015, and is currently indicated as an add-on maintenance treatment for patients aged ≥ 6 years with uncontrolled SA-EP [14]. At the time of this study, mepolizumab was approved for use in patients aged ≥ 18 years. Public reimbursement of mepolizumab, under the Exceptional Access Program within ODB (a categorization that requires specialist prescribing), began in March 2018. As with many biologics, a patient support program (PSP) was implemented to assist patients and caregivers in accessing and managing the medication.

This study used data from the ICES database and ODB program to investigate the real-world impact of mepolizumab on asthma-related outcomes and the associated costs among patients with SA-EP in a real-world population in Ontario, Canada.

## Methods

### Study design

This retrospective, observational study compared cohorts of patients with SA-EP before and after initiating mepolizumab. Patients who initiated mepolizumab during the selection period (February 1, 2016, to March 31, 2019) were identified using the Canadian mepolizumab PSP and linked to the ICES database. Individual patient study outcomes were obtained for the 12 months pre- and post-mepolizumab initiation.

The study comprised one main cohort, the overall PSP population, and two subsets of the overall PSP population: (1) the provincial drug coverage subset (used to track utilization of related medications such as OCS/inhalers independently of reimbursement of mepolizumab through the ODB program); and (2) a second subset of patients who were adherent to treatment (≥ 9 mepolizumab prescriptions within 12 months post-index date).

The ICES captures all health services provided by the Ontario public system, and its data repository consists of de-identified, record-level, coded and linkable health data sets for as many as 13 million Ontarians. The records in ICES data reflect Ontarians’ day-to-day interactions with the healthcare system. These include: (1) physician claims submitted to the Ontario Health Insurance Plan; (2) medical drug claims based on prescriptions submitted to and reimbursed by the ODB program; (3) discharge summaries of hospital stays and ED visits; and (4) claims for home care, long-term care, and more.

### Objectives

The primary objectives of this study were: to describe the demographics and clinical characteristics of real-world patients receiving mepolizumab; to compare asthma-related outcomes before and during mepolizumab use; and to compare asthma-related costs before and during mepolizumab use. The secondary objective was to assess real-world asthma-related outcomes and associated costs for patients who were adherent to mepolizumab treatment (≥ 9 prescriptions in 12 months).

### Study outcomes

Asthma-related real-world outcomes of interest were: asthma exacerbations (defined as outpatient or ED visit with a diagnosis of asthma and at least one dispensing of systemic corticosteroids ± 5 days after the encounter or exacerbations resulting in hospitalization/inpatient hospital admissions with a primary diagnosis of asthma); physician visits (general practitioner [GP], specialist, and other outpatient visits); ED visits; inpatient hospitalizations; OCS use (provincial drug coverage subset only); and short-acting β-agonist (SABA) use (provincial drug coverage subset only).

Real-world outcome costs were: GP cost (asthma-related); specialist cost (asthma-related); ED cost (all-cause); hospital admission cost (all-cause); total cost (excluding all drug costs); drug cost (provincial drug coverage subset only); total cost (including drug cost; provincial drug coverage subset only). When reimbursed by the ODB program after March 2018, the cost of mepolizumab treatment was included in the provincial drug coverage subset costs. Due to resource intensity weighting, costs cannot be accurately estimated from specific hospital admission reasons; thus, any ED visit and inpatient hospitalization costs are not asthma-specific. Cost per service was collected from ICES, which collates data from the Ontario Health Insurance Plan database and covers all publicly funded healthcare billing.

### Patient identification

The Canadian mepolizumab PSP was used to identify patients with mepolizumab utilization, who were then linked to the ICES database. Using deterministic linkage, the patients’ unique Ontario health card numbers connected PSP patients to the provincial ICES dataset which allowed tracking of patients’ healthcare system interactions through both the PSP and the healthcare system. The remaining patients were identified by probabilistic linkage using date of birth, sex, and postal code and linked to a corresponding patient in the ICES Ontario asthma cohort.

### Study population

Patients were included in the study if they had provided informed consent, received ≥ 1 injection of mepolizumab within the selection period, initiated mepolizumab within the selection period, were identified within the ICES database, were ≥ 18 years of age at index, and were active within the ICES database for 1 year pre- and post-mepolizumab index date. Additional inclusion criteria for the provincial drug coverage subset included ≥ 1 ODB asthma claim in both the 12-month pre- and post-mepolizumab analysis periods, and ≥ 1 ODB asthma claim in the 3 months before the pre-mepolizumab period and 3 months after the post-mepolizumab period.

We defined patients adherent to mepolizumab treatment as individuals within the PSP population who received ≥ 9 mepolizumab injections over a 1-year categorization period. This subgroup was further stratified into those who had an eosinophil level ≤ 300 cells/μL (PSP population only), those with ≥ 2 baseline exacerbations and eosinophil level ≥ 300 cells/μL (provincial drug coverage subset only), and those with ≥ 3 baseline exacerbations (provincial drug coverage subset only), where baseline eosinophil counts were recorded as the median eosinophil count in the 12-month pre-mepolizumab period. Additional subgroups were considered but were not evaluated due to low patient numbers.

### Statistical considerations

For the sample size calculation, the minimum detectable reduction in mean exacerbations between pre- and post-mepolizumab initiation periods was used (see Supplementary Table 1, Additional file 1). For all analyses, missing data were reported as their own category, and no imputation was performed. All analyses were conducted using statistical analysis system (SAS) version 9.3 or higher.

Descriptive analyses were performed using the number of patients and percentage for categorical variables, and the mean (standard deviation [SD]) and median (interquartile range [IQR]) for continuous variables. As healthcare resource utilization (HCRU) outcomes and costs data were not continuous and bounded at zero, a distribution other than the normal was assumed. For HCRU, negative binomial distribution was used where variance > mean, or Poisson where mean ≤ variance; gamma distribution was used for cost. Corrections for multiple comparisons were made using a baseline significance level of α = 0.05.

The mean number of events observed in a category was presented for all patients and patients adherent to mepolizumab treatment, and the presence or absence of an event was compared pre- and post-mepolizumab initiation using McNemar’s test. Differences in HCRU counts between pre- and post-mepolizumab initiation (in the overall PSP population and the provincial drug coverage subset) were compared using an adjusted, generalized, linear mixed model with a negative binomial distribution. The model was used to control for covariates and model the HCRU count as the dependent variable, with patient as a random factor to account for the paired nature of the data. Mean costs were compared pre- and post-mepolizumab initiation for all patients (in the overall PSP population and the provincial drug coverage subset) using an adjusted mixed gamma model with patient as a random factor.

### Study ethics

This study complied with all laws regarding patient privacy. No direct patient contact or primary collection of individual human subject data occurred. Patients enrolled or previously enrolled in the Canadian mepolizumab PSP program were given the opportunity to provide informed consent that allowed their patient-level data to be shared with ICES. This was conducted by the program coordinators via email, phone, or in person. All executed versions of the consent form and study protocol were approved by an Institutional Review Board (IRB; Pro00034156). These were first approved in May 2019 and continually reviewed every 12 months by the IRB.

## Results

### Patient demographics and clinical characteristics

#### Overall PSP cohort

**Fig. 1:**
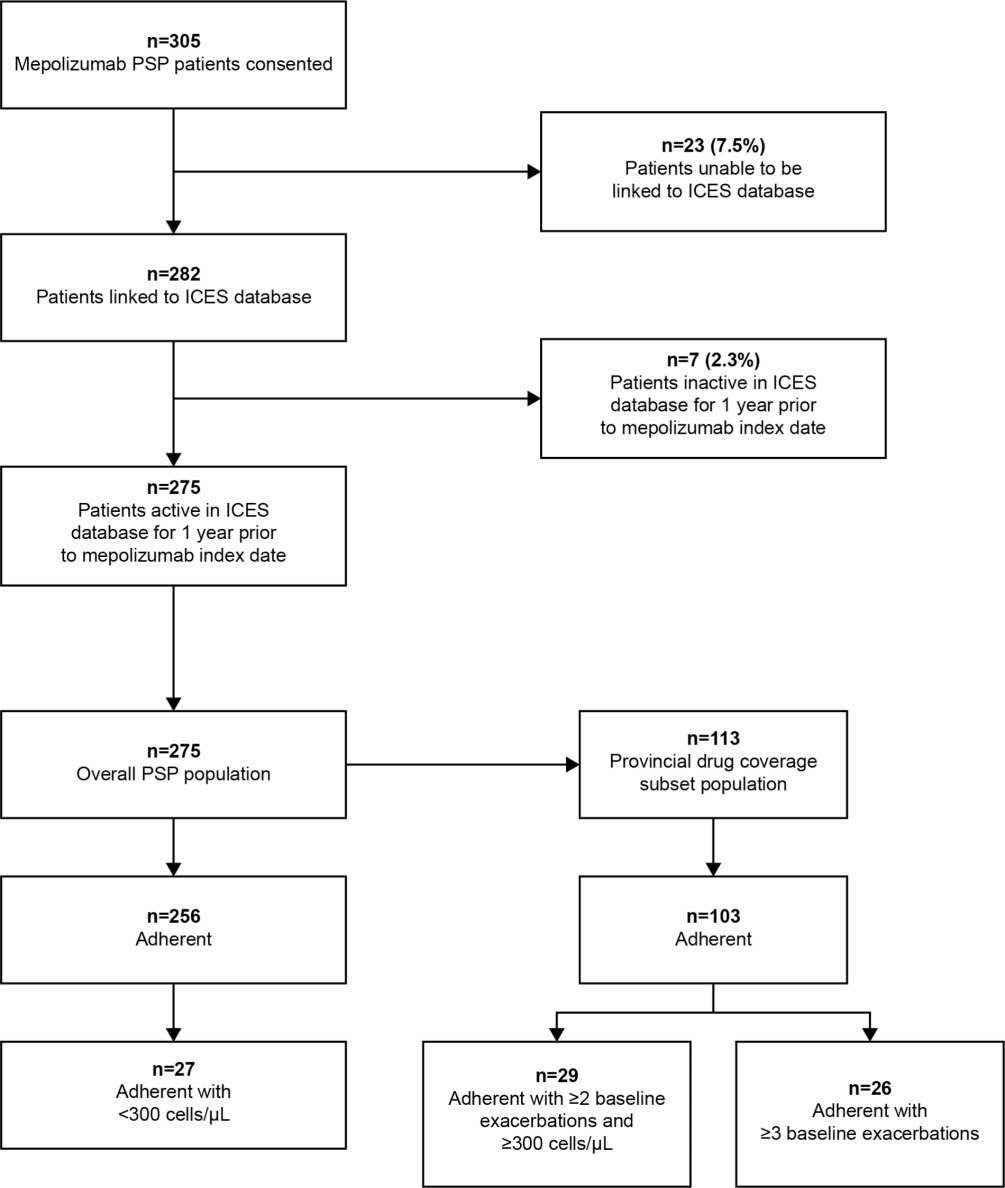
Patient selection Asthma exacerbations were defined as outpatient or emergency department visits with a diagnosis of asthma and at least one dispensing of systemic corticosteroids ± 5 days after the encounter or exacerbations resulting in hospitalization/inpatient hospital admission with a diagnosis of asthma as a primary diagnosis ICES, Institute for Clinical and Evaluative Sciences; PSP, patient support program

The overall PSP cohort included 275 patients (Table 1). The mean (SD) age was 57.6 (13.5) years and 56.0% were female. Most patients were deemed to reside in an urban area (93.1%). Mepolizumab use was distributed evenly amongst income quintiles. Of the 275 patients, 259 (94.2%) had a recorded eosinophil value in the 12-month pre-mepolizumab period. The mean (SD) of the within-patient median eosinophil count level was 540.4 (491.9) cells/μL. Charlson Comorbidity Index (CCI) values were available for 95 patients (34.5%), of which 64 patients were categorized as low (0–1). Of the 275 patients in the overall PSP cohort, 256 (93.1%) were adherent to mepolizumab treatment. Within the adherent subgroup, 27 patients (10.5%) had eosinophil levels < 300 cells/μL (Fig. 1).

**Table 1:** Baseline demographics and clinical characteristics

	Overall PSP cohort (N = 275)		Provincial drug coverage subset (n = 113)	
**Age, years**				
Mean (SD)	57.6 (13.5)		64.6 (12.6)	
Median (IQR)	59.0 (50.0–68.0)		68.0 (59.0–72.0)	
**Female, n (%)**	154 (56.0%)		65 (57.5%)	
**Index year, n (%)**				
2016	70 (25.5%)		25 (22.1%)	
2017	129 (46.9%)		56 (49.6%)	
2018	64 (23.3%)		27–31*	
2019	12 (4.4%)		1–5*	
**Rural residence, n (%)**				
Urban	256 (93.1%)		104 (92.0%)	
Rural	19 (6.9%)		9 (8.0%)	
**Income quintile, n (%)**				
Q1, lowest	50 (18.2%)		29 (25.7%)	
Q2	51 (18.6%)		16 (14.2%)	
Q3	52 (18.9%)		18 (15.9%)	
Q4	64 (23.3%)		24 (21.2%)	
Q5, highest	58 (21.1%)		26 (23.0%)	
**Highest eosinophil count values** ^**†**^	n = 259 (94.2%)		n = 108–112*	
Mean (SD)	925.3 (1275.7)		894.7 (1295.6)	
Median (IQR)	620.0 (400.0–1010.0)		600.0 (325.0–950.0)	
**Median eosinophil count values** ^**‡**^	n = 259 (94.2%)		n = 108–112*	
Mean (SD)	540.4 (491.9)		457.8 (464.1)	
Median (IQR)	400.0 (200.0–700.0)		400.00 (200.0–600.0)	
**Charlson Comorbidity Index, n (%)**	95 (34.5%)		45 (39.8%)	
Low 0–1	64 (23.3%)		24 (21.2%)	
Moderate 2–3	23 (8.4%)		14–18*	
High 4+	8 (2.9%)		3–7*	
Missing	180 (65.5%)		68 (60.2%)	

*Value reported as a range according to ICES reporting standards to reduce the risk of patient re-identification

^†^This represents the patient’s highest eosinophil count in the 12-month pre-mepolizumab period

^‡^This represents the patient’s median eosinophil count in the 12-month pre-mepolizumab period

ICES, Institute for Clinical and Evaluative Sciences; IQR, interquartile range; PSP, patient support program; Q, quintile; SD, standard deviation

#### Provincial drug coverage subset

The provincial drug coverage subset included 113 patients (Table 1). On average, these patients were older than the overall PSP population (mean [SD] age 64.6 [12.6] years), with comparable proportions of patients who were female (57.5%) and who lived in an urban area (92.0%). Of the 113 patients in the provincial drug coverage subset, the mean (SD) of the within-patient median eosinophil count level was 457.8 (464.1) cells/μL. CCI values were available for 45 patients (39.8%), of which 24 patients were categorized as low (0–1). Baseline asthma-related medication use for the provincial drug coverage subset is reported in Table 2. Of the 113 patients in the provincial drug coverage subset, 103 (91.2%) were adherent to mepolizumab treatment. Of these 103 patients within the provincial drug coverage subset adherent subgroup, 29 (28.2%) had ≥ 2 baseline exacerbations and eosinophil levels ≥ 300 cells/μL, and 26 (25.2%) had ≥ 3 baseline exacerbations (Fig. 1).

**Table 2:** Baseline asthma-related medication use in the provincial drug coverage subset (n = 113)

	OCS	SABA	ICS	LABA	SAMA	LAMA	Theophylline	ICS + LABA	LTRA	Biologics^†^
Patients with ≥ 1 claim, n (%)	101 (89.4)	98 (86.7)	49 (43.4)	2–6*	14–18*	85 (75.2)	5–9*	105 (92.9)	1–5*	13 (11.5)
Total claims, n	482	486	187	NA	64	369	43	622	NA	146
Claims per patient, mean (SD)	4.77 (5.1)	4.97 (4.5)	1.65 (2.6)	NA	0.57 (2.1)	3.27 (3.2)	0.38 (1.4)	5.50 (3.1)	NA	1.29 (4.0)
Claims per patient, min–max	0.0–47.0	0.0–20.0	0.0–11.0	NA	0.0–14.0	0.0–14.0	0.0–7.0	0.0–13.0	NA	0.0–21.0

*The value was reported as a range according to ICES reporting standards to reduce the risk of patient re-identification

^†^Only asthma-related biologics were included in the biologics category

ICES, Institute for Clinical and Evaluative Sciences; ICS, inhaled corticosteroid; LABA, long-acting β-agonist; LAMA, long-acting muscarinic antagonist; LTRA, leukotriene receptor antagonist; max, maximum; min, minimum; NA, not applicable; OCS, oral corticosteroid; SABA, short-acting β-agonist; SAMA, short-acting muscarinic antagonist; SD, standard deviation

### HCRU

**Fig. 2:**
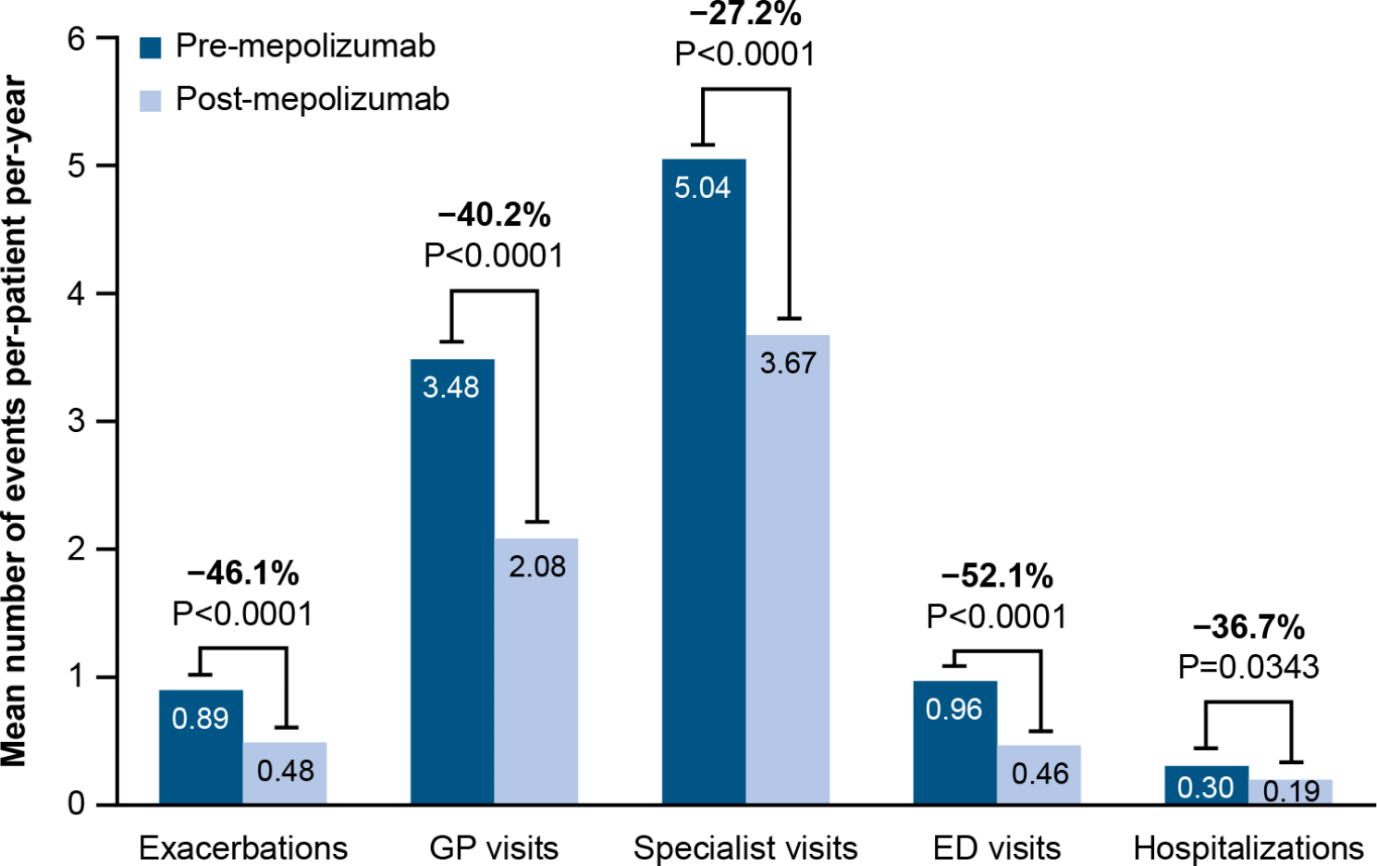
Asthma-related HCRU for patients in the overall PSP population pre- versus post-mepolizumab ED, emergency department; GP, general practitioner; HCRU, healthcare resource utilization; PSP, patient support program

In the overall PSP cohort, mepolizumab use was associated with significant reductions in the mean number of per-patient asthma exacerbations (46.1%, *P* < 0.0001) and all measures of asthma-related HCRU (Fig. 2). The greatest per-patient reduction was in ED visits (52.1%, *P* < 0.0001), with marked decreases in GP visits (40.2%, *P* < 0.001), specialist visits (27.2%, *P* < 0.0001), and hospitalizations (36.7%, *P* = 0.0343). Results for the PSP-adherent subgroup and PSP-adherent (eosinophil level < 300 cells/μL) subgroup were comparable with the overall PSP population (see Supplementary Table 2, Additional file 2).

**Fig. 3:**
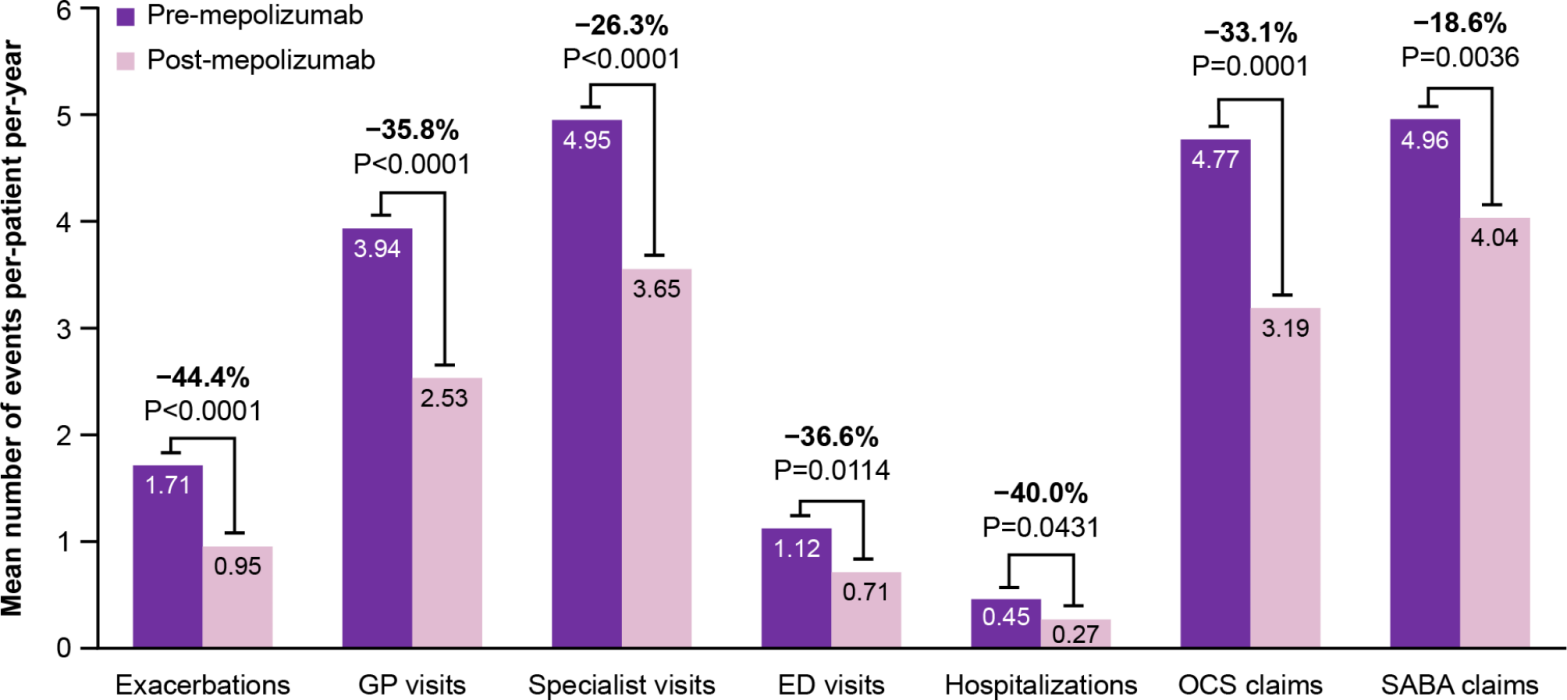
Asthma-related HCRU for patients in the provincial drug coverage subset pre- versus post-mepolizumab ED, emergency department; GP, general practitioner; HCRU, healthcare resource utilization; OCS, oral corticosteroid; SABA, short-acting β-agonist

In the provincial drug coverage subset, statistically significant reductions were observed in the mean number of per-patient asthma exacerbations (44.4%, *P* < 0.0001) and all asthma-related HCRU outcomes (Fig. 3). Decreases were observed in GP visits (35.8%, *P* < 0.0001), specialist visits (26.3%, *P* < 0.0001), ED visits (36.6%, *P* = 0.0114), and hospitalizations (40.0%, *P* = 0.0431). Reductions in mean per-patient claims were observed for OCS (33.1%, *P* = 0.0001) and SABA (18.6%, *P* = 0.0036). Within the provincial drug coverage subset adherent sub-cohort, patients with ≥ 2 baseline exacerbations and eosinophil count of ≥ 300 cells/μL, and patients with ≥ 3 baseline exacerbations, all showed similar results to the provincial drug coverage subset (see Supplementary Table 3, Additional file 3).

### Healthcare costs

**Fig. 4:**
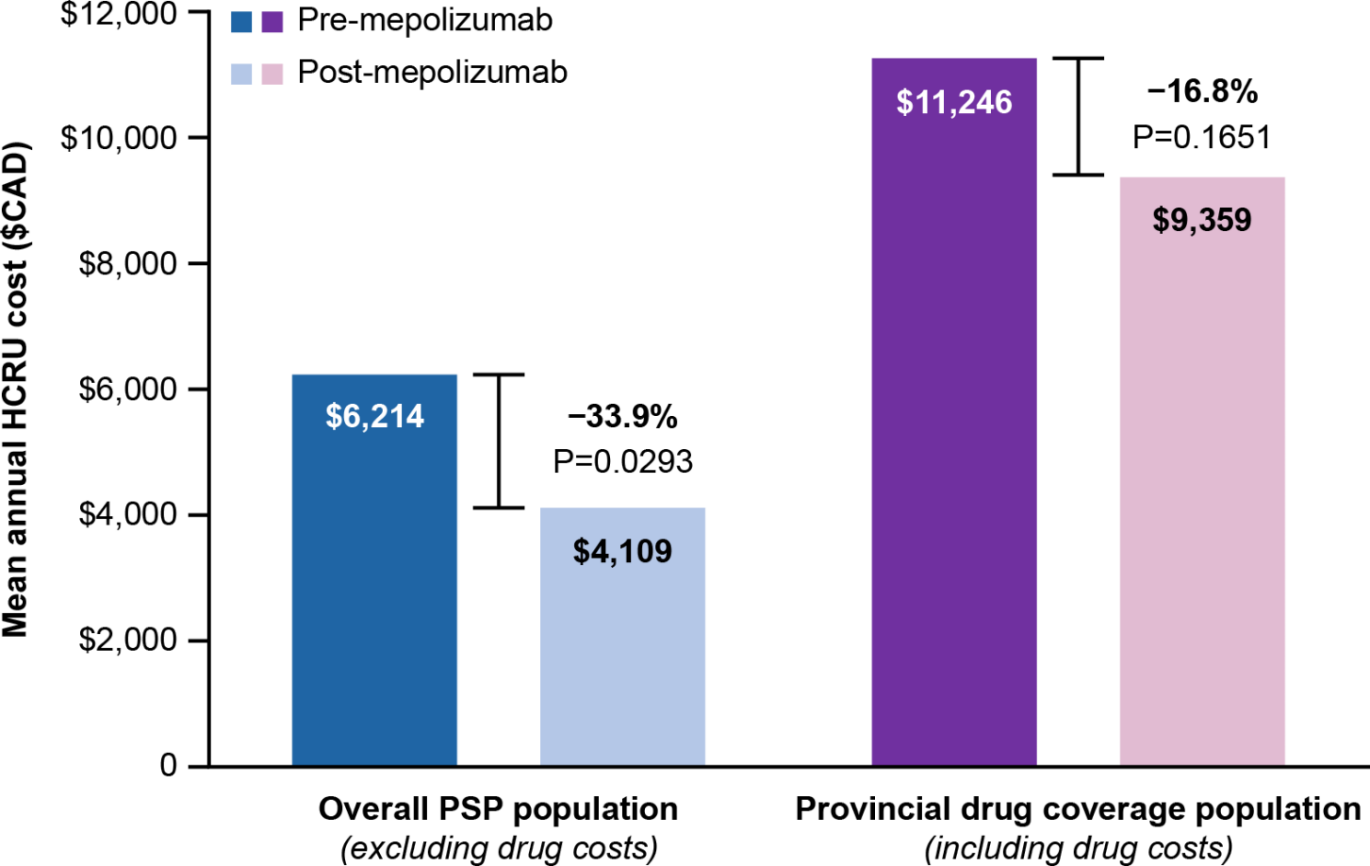
Mean HCRU cost per patient in both populations (pre- versus post-mepolizumab) Includes costs indicated in Table 3 (PSP population) and Table 4 (provincial drug coverage subset population) $CAD, Canadian dollars; HCRU, healthcare resource utilization; PSP, patient support program

In the overall PSP cohort, a reduction in the average cost per patient was seen for all HCRU outcomes after initiating mepolizumab (Table 3). While the all-cause hospitalization costs were reduced by 34%, the result was not statistically significant. For asthma-related GP visits, the average cost per patient was reduced by 37.1% and for asthma-related specialist visits, the average cost per patient was reduced by 36.8%. The mean total HCRU costs (excluding drug costs) in the 12 months before and after initiating mepolizumab were CAD$6,214 versus CAD$4,109 per patient, respectively (33.9% reduction, *P* < 0.05) (Table 3 and Fig. 4).

**Table 3:** Healthcare cost outcomes for the overall PSP cohort (N = 275)

	Mean annual HCRU cost per patient ($CAD)		
Real-world cost outcome	Pre-mepolizumab	Post-mepolizumab	% change
GP visit (asthma-related)	$83.55	$52.54	–37.1***
Specialist visit (asthma-related)	$390.74	$246.82	–36.8***
ED visits (all-cause)	$602.69	$447.82	–25.7**
Hospital admission (all-cause)^†^	$4,372.92	$2,885.32	–34.0
HCRU (excluding drug cost)^‡^	$6,214.42	$4,109.12	–33.9*

*P < 0.05

**P < 0.01

***P < 0.001

^†^Due to resource intensity weighting, costs cannot be accurately estimated from specific hospital admission reasons; thus, costs are not asthma-specific

^‡^HCRU includes all factors listed in table as well as other asthma-related physician costs for shadow billings, where the location was other than home, office, or phone

$CAD, Canadian dollars; ED, emergency department; GP, general practitioner; HCRU, healthcare resource utilization; PSP, patient support program

In the provincial drug coverage subset, a directionally similar reduction in average cost per patient was observed for all HCRU outcomes except for asthma-related drug cost (Table 4). In this subset however, only asthma-related specialist visits reached statistical significance. The mean total HCRU costs (excluding drug costs) in the 12 months before and after initiating mepolizumab were CAD$7,566 versus CAD$5,206 per patient, respectively (31.2% reduction) (Table 4). Mean per-patient asthma-related drug costs (including mepolizumab) increased from CAD$3,680 before mepolizumab to CAD$4,153 after mepolizumab (11.4%), contributing to mean total HCRU costs (including drug costs) of CAD$11,246 versus CAD$9,359 per patient (16.8% reduction), respectively (Fig. 4).

**Table 4:** Healthcare cost outcomes for the provincial drug coverage subset (n = 113)

	Mean annual HCRU cost per patient ($CAD)		
Real-world cost outcome	Pre-mepolizumab	Post-mepolizumab	% change
GP visit (asthma-related)	$98.97	$70.02	–29.3
Specialist visit (asthma-related)	$400.01	$259.57	–34.7***
ED visits (all-cause)	$845.35	$724.50	–14.3
Hospital admission (all-cause)^†^	$5,381.49	$3,569.75	–33.7
HCRU (excluding drug cost)^‡^	$7,565.79	$5,205.87	–31.2
Drug cost (asthma-related)	$3,679.92	$4,152.60	+ 11.4
HCRU (including drug cost)	$11,245.71	$9,358.47	–16.8

***P < 0.001

^†^Due to resource intensity weighting, costs cannot be accurately estimated from specific hospital admission reasons; thus, costs are not asthma-specific

^‡^HCRU includes all factors listed in table as well as other asthma-related physician costs for shadow billings, where the location was other than home, office, or phone

$CAD, Canadian dollars; ED, emergency department; GP, general practitioner; HCRU, healthcare resource utilization

## Discussion

This observational real-world study showed that mepolizumab use in real-world clinical practice in Ontario, Canada, was associated with a reduction in HCRU, including exacerbations and hospitalizations. These results are aligned with other studies assessing real-world outcomes of mepolizumab. The REALITI-A study assessed real-world outcomes of mepolizumab in 822 patients globally (57 patients in Canada) and showed significant reductions in exacerbations and daily OCS dose [6]. As REALITI-A included patients from several different countries with differing reimbursement criteria, costs of HCRU were not included in the analysis. A US claims database study [15] that assessed the impact of mepolizumab on OCS use, asthma exacerbations, and asthma exacerbation-related costs in a real-world setting found similar results, with a 51.5% reduction in asthma exacerbation-related costs versus the pre-mepolizumab period.

Although REALITI-A, the aforementioned US claims study, and the current study all demonstrated an association between mepolizumab use and decreased asthma exacerbations (REALITI-A: 71%; US study: 45.5%; current study: 46.1%), there were differences in the definition of exacerbations that may have resulted in differences in the magnitude of reduction seen [6, 15]. In REALITI-A, exacerbations were defined as a deterioration in asthma requiring systemic corticosteroids for ≥ 3 days or a single systemic administration of corticosteroids and/or hospitalization and/or ED visit. By contrast, the US study had a more stringent definition of exacerbations (one outpatient or ED claim with a diagnosis of asthma and at least one claim for a systemic corticosteroid ± 5 days of the encounter, or exacerbations resulting in hospitalization) that was comparable with the definition used in the current study, and the similarities in exacerbation criteria are reflected in the similar levels of exacerbation reduction observed.

As PSP support (nursing support, patient education and injection services, pharmacy services [including counselling and medication delivery], reimbursement navigation, and financial assistance) was provided to all patients, the positive outcome effects observed following mepolizumab initiation are a result of a combination of mepolizumab efficacy and any support-related factors; it is not possible to quantify the extent to which the PSP support contributed to the patients’ outcomes. Patients receiving mepolizumab injections from their healthcare provider may have visited their clinic at monthly intervals to receive treatment. This provided patients with additional touchpoints with healthcare professionals that may have resulted in early identification and treatment of asthma worsening, potentially leading to fewer exacerbations.

Only a small percentage of patients were less than optimally adherent to mepolizumab treatment and received fewer than 9 of 12 scheduled injections. This contrasts with low adherence figures typically seen with inhaled medications in asthma [16] and is noteworthy given that most injections would have been clinic-based rather than patient self-administered. Better outcomes were seen in the adherent group, although the number of patients who were sub-optimally adherent to mepolizumab treatment was small. Such an outcome likely reflects the efficacy of mepolizumab and would suggest that strategies to improve adherence would improve the efficacy of the intervention [17]. We cannot rule out the possibility that patients who were poorly adherent to mepolizumab treatment were less responsive and that missed doses were the consequence rather than the cause of poor asthma outcomes. We also note that the presented data do not allow us to explore the temporal relationship between exacerbations and missed mepolizumab visits.

A strength of this study is that it reflects the real-world effect of mepolizumab on patients with asthma, complementing clinical trial evidence. Furthermore, few exclusion criteria were applied, reflecting diverse and unselected populations in Ontario who may not otherwise be included in clinical trials.

There are several limitations to note within this study. First, the database used provided data only for Ontario residents with health coverage, so estimates of HCRU may not be generalizable to individuals in other provinces or those with different or no health insurance. Second, use of an administrative database may also have introduced measurement errors such as inaccurate coding and data entry. Furthermore, the provincial drug coverage dataset only includes publicly reimbursed drug claims largely for patients aged ≥ 65 years; therefore, patients < 65 years old and those with cash and private claims may not be included in this subset. Additionally, hospital costs were calculated using a weighted average resource intensity method that could potentially underestimate outliers. For patients with hospitalization, HCRU and associated costs were captured for all healthcare touchpoints which could not be separated for asthma versus other conditions. A further limitation is that the study design did not account for asthma progression; however, this is similar to the recent US claims study which also noted that exacerbations remained constant independent of treatment [15]. The current study did not distinguish between OCS for maintenance use and episodically for acute exacerbation. Consequently, if a patient had an outpatient/ED visit with an asthma diagnosis and OCS dispensing within 5 days, that was considered an asthma exacerbation. Nevertheless, the reduction in exacerbations shown in this study is likely reliable as reductions were observed across all outcomes. Despite these limitations, this real-world study reiterates the value of mepolizumab treatment and its ability to reduce the burden of severe asthma on patients and the healthcare system, as similarly seen in the clinical trial setting.

## Conclusion

In conclusion, this study has shown that treatment with mepolizumab resulted in reduced asthma exacerbations and decreased HCRU and associated costs in Canadian patients in Ontario with SA-EP. This could translate to greater asthma control, leading to improved quality of life and productivity.

### Electronic supplementary material

Below is the link to the electronic supplementary material.

**Additional file 1: Supplementary Table 1.** Sample sizes to detect a minimum detectable reduction in mean exacerbation rate pre- and post-mepolizumab

**Additional file 2: Supplementary Table 2.** Mean number of real-world outcomes for the PSP-adherent subpopulations following mepolizumab initiation

**Additional file 3: Supplementary Table 3.** Mean number of real-world outcomes for the provincial drug coverage adherent subpopulations following mepolizumab initiation

## Data Availability

The dataset from this study is held securely in coded form at ICES. While legal data sharing agreements between ICES and data providers (e.g., healthcare organizations and the government) prohibit ICES from making the dataset publicly available, access may be granted to those who meet pre-specified criteria for confidential access, available at https://www.ices.on.ca/use-ices-data/working-with-ices-data/ (accessed on 05 September 2023) (email: das@ices.on.ca). The full dataset creation plan and underlying analytic code are available from the authors upon request, understanding that the computer programs may rely upon coding templates or macros that are unique to ICES and are, therefore, either inaccessible or may require modification.

## Abbreviations

CAD$: Canadian dollars
CCI: Charlson Comorbidity Index
CSE: Clinically significant exacerbation
ED: Emergency department
GP: General practitioner
HCRU: Healthcare resource utilization
ICES: Institute for Clinical and Evaluative Sciences
ICS: Inhaled corticosteroid
IL5: Interleukin 5
IQR: Interquartile range
IRB: Institutional Review Board
LABA: Long-acting β-agonist
LAMA: Long-acting muscarinic antagonist
LTRA: Leukotriene receptor antagonist
max: Maximum
mepo: Mepolizumab
min: Minimum
NA: Not applicable
OCS: Oral corticosteroid
ODB: Ontario Drug Benefit
PSP: Patient support program
Q: Quintile
SA-EP: Severe asthma with an eosinophilic phenotype
SABA: Short-acting β-agonist
SAMA: Short-acting muscarinic antagonist
SAS: Statistical analysis system
SD: Standard deviation
UK: United Kingdom
US$: United States dollars
US: United States
