# Supplementary material for: Real-world outcomes of mepolizumab for the treatment of severe eosinophilic asthma in Canada: an observational study
Source: Allergy Asthma Clin Immunol. 2024 Feb 4;20:11. doi: 10.1186/s13223-023-00863-7 (PMC10838436; doi:10.1186/s13223-023-00863-7)
Supplement: Supplementary file 1 — Additional file 1: Supplementary Table 1. Sample sizes to detect a minimum detectable reduction in mean exacerbation rate pre- and post-mepolizumab [file 13223_2023_863_MOESM1_ESM.docx]

**Additional file 1
Supplementary Table 1.** Sample sizes to detect a minimum detectable reduction in mean exacerbation rate pre- and post-mepolizumab

| **Sample size (n)** | **Minimum detectable reduction (%)** |
| --- | --- |
| 100 | 32–36 |
| 125 | 29–32 |
| 135 | 28–31 |
| 150 | 27–29 |
| 175 | 25–27 |
| 200 | 22–25 |
| 225 | 19–24 |
| 250 | 21–23 |
| 300 | 19–21 |
