# Supplementary material for: Real-world outcomes of mepolizumab for the treatment of severe eosinophilic asthma in Canada: an observational study
Source: Allergy Asthma Clin Immunol. 2024 Feb 4;20:11. doi: 10.1186/s13223-023-00863-7 (PMC10838436; doi:10.1186/s13223-023-00863-7)
Supplement: Supplementary file 2 — Additional file 2: Supplementary Table 2. Mean number of real-world outcomes for the PSP-adherent subpopulations following mepolizumab initiation [file 13223_2023_863_MOESM2_ESM.docx]

**Additional file 2**

**Supplementary Table 2.** Mean number of real-world outcomes for the PSP-adherent subpopulations following mepolizumab initiation

|  | **Overall PSP population**  **(N = 275)** | | **PSP-adherent subpopulation**  (≥9 treatments in 12 months) | | | |
| --- | --- | --- | --- | --- | --- | --- |
|  |  |  | **All patients (n = 256)** | | **<300 cells/µL (n = 27)** | |
|  | **Pre-mepo** | **Post-mepo** | **Pre-mepo** | **Post-mepo** | **Pre-mepo** | **Post-mepo** |
| **Exacerbations, n** | 0.89 | 0.48 | 0.89 | 0.47 | 1.19 | 0.93 |
|  | –46.1%*** | | –47.2%*** | | –21.9% | |
| **Asthma-related visits, n** | | | | | | |
| GP | 3.48 | 2.08 | 3.59 | 2.10 | 3.04 | 2.63 |
|  | –40.2%*** | | –41.5%*** | | –13.5% | |
| Specialist | 5.04 | 3.67 | 5.12 | 3.69 | 5.33 | 3.67 |
|  | –27.2%*** | | –27.9%*** | | –31.1% | |
| Emergency department | 0.96 | 0.46 | 0.96 | 0.45 | 1.22 | 0.67 |
|  | –52.1%*** | | –53.1%*** | | –45.1% | |
| Inpatient hospitalization | 0.30 | 0.19 | 0.31 | 0.19 | NA | NA |
|  | –36.7%* | | –38.7%* | | NA | |

**P*<0.05
***P*<0.01
****P*<0.0001
GP, general practitioner; mepo, mepolizumab; NA, not available; PSP, patient support program
