# Supplementary material for: Real-world outcomes of mepolizumab for the treatment of severe eosinophilic asthma in Canada: an observational study
Source: Allergy Asthma Clin Immunol. 2024 Feb 4;20:11. doi: 10.1186/s13223-023-00863-7 (PMC10838436; doi:10.1186/s13223-023-00863-7)
Supplement: Supplementary file 3 — Additional file 3: Supplementary Table 3. Mean number of real-world outcomes for the provincial drug coverage adherent subpopulations following mepolizumab initiation [file 13223_2023_863_MOESM3_ESM.docx]

**Additional file 3
Supplementary Table 3.** Mean number of real-world outcomes for the provincial drug coverage adherent subpopulations following mepolizumab initiation

|  | **Provincial drug coverage subset population**  **(n = 113)** | | **Provincial drug coverage subset adherent subpopulation**  (≥9 treatments in 12 months) | | | | | |
| --- | --- | --- | --- | --- | --- | --- | --- | --- |
|  |  |  | **All patients**  **(n = 103)** | | **≥2 baseline exacerbations and ≥300 cells/µL (n = 29)** | | **≥3 baseline exacerbations**  **(n = 26)** | |
|  | **Pre-mepo** | **Post-mepo** | **Pre-mepo** | **Post-mepo** | **Pre-mepo** | **Post-mepo** | **Pre-mepo** | **Post-mepo** |
| **Exacerbations, n** | 1.71 | 0.95 | 1.76 | 1.00 | 4.07 | 1.69 | 4.85 | 2.27 |
|  | –44.4%*** | | –43.2%** | | –58.5% | | –53.2% | |
| **Asthma-related visits, n** | | | | | | | | |
| GP | 3.94 | 2.53 | 4.07 | 2.59 | 6.45 | 3.41 | 7.62 | 4.12 |
|  | –35.8%*** | | –36.4%*** | | –47.1% | | –45.9% | |
| Specialist | 4.95 | 3.65 | 5.01 | 3.63 | 6.34 | 4.52 | 6.85 | 4.42 |
|  | –26.3%*** | | –27.5%*** | | –28.7% | | –35.5% | |
| Emergency department | 1.12 | 0.71 | 1.17 | 0.72 | 2.10 | 1.45 | 3.23 | 2.00 |
|  | –36.6%* | | –38.5%* | | –31.0% | | –38.1% | |
| Inpatient hospitalization | 0.45 | 0.27 | 0.49 | 0.30 | 0.90 | 0.69 | 1.42 | 0.96 |
|  | –40.0%* | | –38.8%* | | –23.3% | | –32.4% | |
| **Treatment claims, n** | | | | | | | | |
| OCS | 4.77 | 3.19 | 4.85 | 3.21 | 6.10 | 3.83 | 6.65 | 4.38 |
|  | –33.1%*** | | –33.8%** | | –37.2% | | –34.1% | |
| SABA | 4.96 | 4.04 | 5.11 | 4.08 | 6.10 | 4.59 | 6.50 | 4.81 |
|  | –18.6%** | | –20.2%** | | –24.8% | | –26.0% | |

**P*<0.05
***P*<0.01
****P*<0.0001
GP, general practitioner; mepo, mepolizumab; OCS, oral corticosteroid; SABA, short-acting β-agonist
